# Polyploid evolution and Pleistocene glacial cycles: A case study from the alpine primrose *Primula marginata* (Primulaceae)

**DOI:** 10.1186/1471-2148-12-56

**Published:** 2012-04-24

**Authors:** Gabriele Casazza, Laura Granato, Luigi Minuto, Elena Conti

**Affiliations:** 1 DISTAV, University of Genova, Corso Dogali 1M, I-16136, Genova, Italy; 2 Institut für Systematische Botanik, Universität Zürich, Zollikerstrasse 107, CH-8008, Zürich, Switzerland

**Keywords:** Allopolyploidy, Flow cytometry, Non-adaptive processes, Phylogeny, *Primula marginata*, Alps, Refugia, Pleistocene, Climate change, Taxonomy

## Abstract

**Background:**

Recent studies highlighted the role of Pleistocene climatic cycles in polyploid speciation and of southern Alpine refugia as reservoirs of diversity during glacial maxima. The polyploid *Primula marginata*, endemic to the southwestern Alps, includes both hexaploid and dodecaploid cytotypes that show no ecological or morphological differences. We used flow cytometry to determine variation and geographic distribution of cytotypes within and between populations and analyses of chloroplast (cp) and nuclear ribosomal (nr) DNA sequences from the Internal Transcribed Spacer (ITS) region to infer the evolutionary history of the two cytotypes and the auto- vs. allopolyploid origin of dodecaploid populations.

**Results:**

We did not detect any intermediate cytotypes or variation of ploidy levels within populations. Hexaploids occur in the western and dodecaploids in the eastern part of the distributional range, respectively. The cpDNA and nrDNA topologies are in conflict, for the former supports shared ancestry between *P. marginata* and *P. latifolia*, while the latter implies common origins between at least some ITS clones of *P. marginata* and *P. allionii*.

**Conclusions:**

Our results suggest an initial episode of chloroplast capture involving ancestral lineages of *P. latifolia* and *P. marginata*, followed by polyploidization between *P. marginata*-like and *P. allionii*-like lineages in a southern refugium of the Maritime Alps. The higher proportion of ITS polymorphisms in dodecaploid than in hexaploid accessions of *P. marginata* and higher total nucleotide diversity of ITS clones in dodecaploid vs. hexaploid individuals sequences are congruent with the allopolyploid hypothesis of dodecaploid origin.

## Background

Polyploid speciation, characterized by a heritable increase in the copy number of the nuclear genome, has played a key role in plant evolution [1-7]. Indeed, recent analyses estimated that all seed plants trace their origins to a few polyplodization events [1] and that 15% of speciation events involved polyplodization [8]. Occurring either between (i.e., allopolyploidization) or within the same species (i.e., autopolyploidization), polyploidization often involves hybridization between genetically differentiated populations, also in the latter case [9,10]. Polyploids can form repeatedly from different populations of the same progenitor species (i.e., polyphyletic origins), a pattern that further contributes to the increase of genetic variability typical of polyploids, as compared to relatives at lower ploidy levels [11,12]. Additionally, the higher genetic variability and the epigenetic alterations usually associated with polyploids have been invoked to explain their wider ecological tolerance and adaptive success [13-16].

The effects of Pleistocene glacial cycles on alpine and arctic regions presumably played a key role in promoting polyploid speciation. According to the secondary contact model [17,18], glacial advancement during glacial maxima fragmented once continuous populations that then differentiated in isolation. Upon glacial retreat, these partially differentiated populations may have come into contact again, occasionally giving origin to hybrids that were then stabilized by polyploidization. The higher genetic variability and adaptive potential of the polyploids would have increased their ability to become established in newly available areas following glacial retreat, thus explaining the higher frequency of polyploids at higher altitudes and latitudes [19]. Molecular investigations have since provided evidence supporting speciation via secondary contact in several alpine/arctic species (e.g., [20-24]).

**Figure 1:**
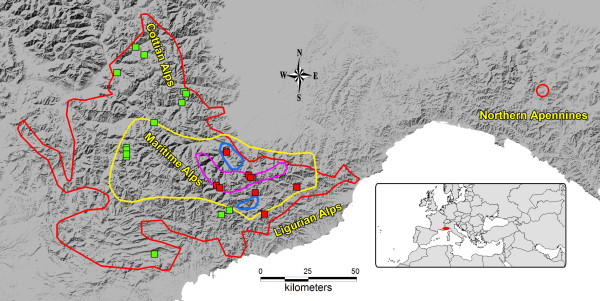
**Geographic distribution of*P. marginata, P. allionii*,*P. hirsuta*and*P. latifolia*in the SW Alps.** Areas of distribution of *P. marginata* (red line), *P. allionii* (blue line), *P. hirsuta* (pink line) and *P. latifolia* (yellow line). Cytotype distribution of *P. marginata* according to Kress (1969): green squares = hexaploids; red squares = dodecaploids. The major mountain chains in the distributional areas of the species are labeled (Cottian, Maritime and Ligurian Alps within the Western Alps; Northern Apennines).

Polyploidization within the context of the Pleistocene glacial cycles has been proposed as the main evolutionary process driving the diversification of at least two sections in the alpine/arctic genus *Primula *[25]: *Primula* sect. *Aleuritia *[23,26-28] and *Primula* sect. *Auricula *[25,29-32]. The latter section includes *P. marginata* Curtis, endemic to the south-western Alps [25]. Populations at both the hexaploid (2*n* = 6*x* = 62, 66) and dodecaploid (2*n* = 12*x* = 120–128) levels have been reported in *P. marginata* (Figure 1)*,* the former occurring primarily in the western (i.e., from the Cottian to the south-western Maritime Alps) and the latter in the eastern part of its range (i.e., from the south-eastern Maritime and Ligurian Alps to the Apennines [31,33,34]). Recently, new disjoint populations of undetermined ploidy level were described in the northern Apennines [35,36].

Both allo- and auto-polyploid hypotheses have been suggested to explain the origin of the 12x populations of *P. marginata.* Favarger [33] proposed that the dodecaploid populations derived from hybridization between hexaploid individuals of *P. marginata* and *P. latifolia* Lapeyr.*,* probably owing to the frequent co-occurrence of the two species. On the contrary, Kress [34] favored an autopolyploid origin, owing to the absence of any morphological and ecological differences distinguishing the two ploidy races. In nature, *P. marginata* is known to hybridize with *P. latifolia *[30] and *P. allionii* Loisel. [25], both co-occurring species of sect. *Auricula* (Figure 1). In cultivation, *P. marginata* has also occasionally formed hybrids with *P. hirsuta* All., another co-occurring species of sect. *Auricula*, but natural hybrids between these two species are unknown [25].

The present study is aimed at clarifying the geographic distribution of *P. marginata* cytotypes and their evolutionary history by determining the ploidy levels of multiple populations across the entire species range and investigating their phylogenetic relationships with increased infra-specific sampling. More specifically, we are asking the following questions: 1) What is the variation of ploidy levels in *P. marginata*? Are there any intermediate cytotypes? 2) Do hexaploid and dodecaploid populations of *P. marginata* occupy distinct areas of the species range, as suggested by published cytological investigations [31,34]? 3) Do hexaploid and dodecaploid populations form reciprocally monophyletic groups? 4) Are the dodecaploid populations of allopolyploid or autopolyploid origin, as proposed by Favarger [33] and Kress [34], respectively? The results of our study have broader implications to elucidate the role of polyploidization in areas affected by Pleistocene glacial cycles.

## Results

### Ploidy level survey

Flow cytometry analyses identified two groups of populations based on relative fluorescence intensity (Table 1): one ranging from 165 to 194 (hexaploids) and the other ranging from 322 to 373 (dodecaploids). The analysis yielded high-resolution histograms, with average CV of 4.52% (range 3.54-5.26%) for hexaploids and 4.24% (range 3.19-5.38%) for dodecaploids. The investigation of 100 individuals revealed no intermediate ploidy levels. In addition, we found no variation of ploidy levels within the same population.

**Table 1:** Results of flow cytometry analyses

** Population**	**code**	**N**	**Coefficient of variation %**	**Relative fluorescence intesity**
HEXAPLOID	**mar_1**	5	4.83 (0.35)	165.33 (3.30)
	**mar_2**	8	3.99 (0.49)	177.00 (2.89)
	**mar_3**	4	3.64 (0.18)	173.89 (2.25)
	**mar_4**	6	5.26 (0.73)	166.48 (5.85)
	**mar_5**	5	5.16 (0.76)	171.28 (4.93)
	**mar_6**	8	4.67 (0.42)	174.27 (2.89)
	**mar_7**	6	3.54 (0.30)	194.21 (2.54)
	**mar_8**	9	4.89 (0.16)	164.42 (1.93)
	**mar_9**			
Total hexaploid		51	4.52 (0.74)	173.19 (9.56)
DODECAPLOID	**mar_10**	6	5.38 (0.93)	333.97 (9.09)
	**mar_11**	5	4.69 (0.23)	324.62 (5.15)
	**mar_12**	5	4.86 (0.58)	321.78 (5.49)
	**mar_13**	8	4.16 (0.36)	324.90 (9.60)
	**mar_14**	5	3.46 (0.25)	353.78 (5.43)
	**mar_15**	5	3.86 (0.27)	359.44 (5.49)
	**mar_16**	3	4.43 (0.17)	356.47 (7.17)
	**mar_17**	5	3.19 (0.20)	373.15 (4.34)
	**mar_18**	7	3.75 (0.48)	367.04 (12.06)
Total dodecaploid		49	4.24 (0.82)	343.93 (21.14)

Populations codes (as in Table 4), numbers of individual analyzed (N), coefficient of variation in percentage and relative fluorescence intensity, with standard deviation in parentheses, are reported.

### cpDNA phylogeny

**Figure 2:**
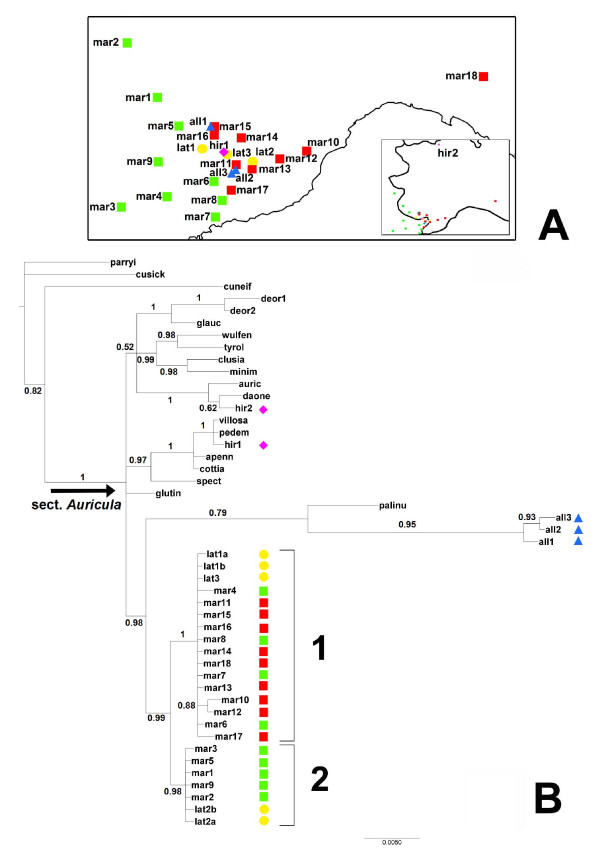
**Geographic distribution and cpDNA tree of*P. marginata*and relatives. A)** Geographic distribution of sampled populations of *P. marginata, P. allionii*, *P. latifolia* and *P. hirsuta* in the Western Alps and Northern Apennines; **B)** Bayesian 50% majority-rule consensus tree of *Primula* sect. *Auricula* accessions; Posterior Probability (PP) values are indicated. *P. marginata* hexaploids = green squares; *P. marginata* dodecaploids = red squares; *P. allionii* = blue triangles; *P. latifolia* = yellow circles; *P. hirsuta* = pink diamonds. Ploidy levels according to flow cytometry data of the present study (Table 1). Population codes as in Table 4 and Additional file 1.

The aligned cpDNA data matrix consisted of 2334 characters (including 19 gaps coded as binary characters), of which 215 were variable (corresponding to 9% of the matrix) and 135 parsimony informative (corresponding to 6% of the matrix). The MP yielded 40 most parsimonious trees, with a consistency index (CI) of 0.79, a retention index (RI) of 0.87 and a rescaled consistency index (RC) of 0.69. In both BI and MP majority-rule consensus trees, all *P. marginata* accessions formed a strongly supported clade with *P. latifolia* accessions (Posterior Probability: PP = 0.99, Bootstrap Support: BS = 76%; Figure 2B, Additional file 1: Figure S1)*.* The clade was divided into two parts (1 and 2 in Figure 2B, Additional file 1: Figure S1). Clade 1 (PP = 1, BS = 92%) comprised both hexaploid (mar4,6-8) and dodecaploid (mar10-18) populations of *P. marginata,* located in the south-eastern part of its range, and two populations of *P. latifolia* (lat1,3). In clade 1 of the Bayesian phylogeny (Figure 2B), the two individuals representing populations mar10 and mar12, located on the eastern border of the range, were sister to each other (PP = 0.88). Clade 2 (PP = 0.98, BS = 71%) comprised only hexaploid populations of *P. marginata* (mar1-3,5,9), geographically located in the north-western part of its range (Figure 2A), together with two individuals of a population of *P. latifolia* (lat2; Figure 2B, Additional file 1: Figure S1).

The three accessions of *P. allionii* formed a monophyletic group (PP = 0.95, BS = 81%), subtended by a long branch, that was sister to *P. palinuri* in the Bayesian tree (PP = 0.79; Figure 2B) and formed polytomy with the same species and the *P. marginata/P. latifolia* clade in the MP tree (Additional file 1: Figure S1). All analyses supported a clade formed by *P. allionii, P. palinuri, P. latifolia* and *P. marginata* (PP = 0.98, BS = 66%; Figure 2B, Additional file 1: Figure S1). The two samples of *P. hirsuta*, the fourth species that geographically co-occurs with *P. marginata*, were not sister to each other: hir1 formed a well-supported clade with *P. villosa* and *P. pedemontana* (PP = 1, BS = 90%); hir2 was included in a statistically strong clade with *P. auricula* and *P. daonensis* (PP = 1; BS = 100%; Figure 2B, Additional file 1: Figure S1).

### nrDNA variation

Between seven and 14 ribotypes per individual of *P. marginata* were detected, for a total of 147 nrDNA sequences. The matrix included a total of 670 characters (662 aligned bps: ITS1 = 282 bps, 5.8S = 158 bps, ITS2 = 222 bps; and eight binary coded gaps). We found a total of 61 substitutions and two single-nucleotide gaps in the 5.8S region; 126 substitutions and nine gaps, of which five were apomorphic, in the ITS1 region; and 102 substitutions and four gaps, of which two were apomorphic, in the ITS2 region. The average G-C content was 53.5%. Only one (3SEQ) of the seven methods implemented in the analysis of recombination using RDP3 detected one statistically significant recombination event (Additional file 1: Table S6), indicating that recombinant ITS clones did not affect our analyses.

**Figure 3:**
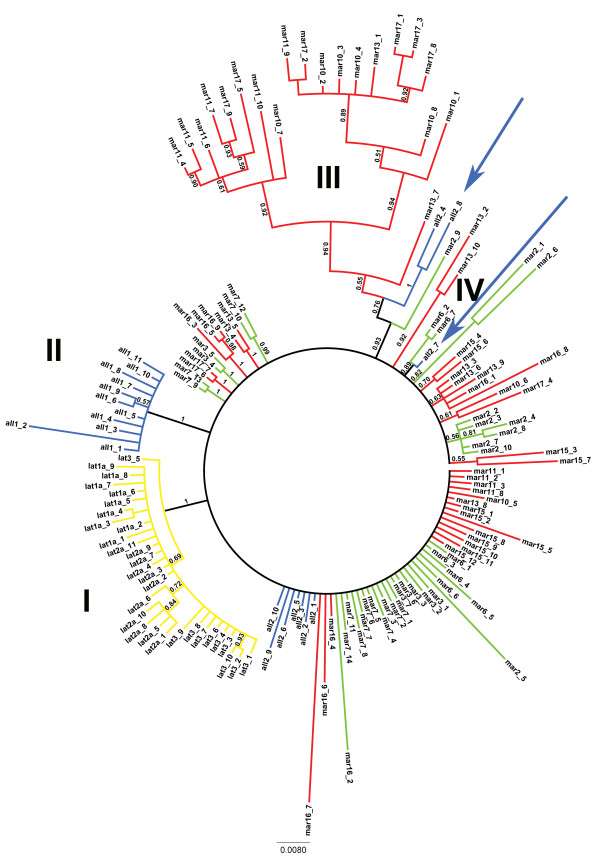
**Bayesian 50% majority-rule consensus tree of cloned ITS sequences.** Bayesian tree inferred from cloned ITS sequences of *P. marginata*, *P. allionii* and *P. latifolia*. Posterior probability values are reported. The colors distinguish species and ploidy levels: *P. marginata* hexaploids = green. *P. marginata* dodecaploids = red. *P. allionii* = blu. *P. latifolia* = yellow. Accessions codes as in Table 4; clones are numbered progressively for each accession. Clade I: all sequences of *P. latifolia*; Clade II: all sequences from all1 population of *P. allionii*; Clade III: sequences from dodecaploid populations mar10, mar11, mar13, mar17 of *P. marginata*; Clade IV: sequences from the hexaploid mar6 population of *P. marginata* and all2 population of *P. allionii*. The two blue arrows indicate the ITS clones from individual all2 of *P. allionii* that form clades with ITS clones from *P. marginata*.

Two hundred and ninety-seven of the 670 characters were variable, of which 127 were parsimony informative, corresponding to 18.95% of the aligned length. MP analyses of the nrDNA matrix yielded 7 most parsimonious trees, with a CI of 0.66, RI of 0.85 and a RC of 0.56. The consensus trees resulting from both BI and MP analyses of all cloned ribotypes were largely unresolved, with the following main exceptions (Figure 3, Additional file 1: Figure S2): I) a clade formed by all sequences of all populations of *P. latifolia* (PP = 1, BS = 97%); II) a clade comprising all sequences from the all1 population of *P. allionii* (PP = 1, BS = 89%); III) a clade formed by sequences from four dodecaploid, western populations of *P. marginata* (mar10,11,13,17; PP = 0.55, BS = 52%); in the BI tree this clade additionally clusters with two sequences from one population of *P. allionii* (all2; PP = 0.76) and one sequence from one hexaploid eastern population of *P. marginata* (mar2; PP = 0.93); IV) a small clade including two sequences from the hexaploid mar6 population and one sequence from the all2 population (PP = 0.89, BS = 64%).

The matrix of cloned ITS sequences included a total of 31 additive polymorphisms in seven sites: 22 (corresponding to 70.97% of the total number) occurred in the dodecaploid accessions of *P. marginata*, whereas only six (19.35%) belonged to *P. marginata* hexaploids and three (9.68%) to *P. allionii* (which is consistently hexaploid). Conversely, *P. latifolia* sequences did not display any additive polymorphisms (Table 2).

**Table 2:** **Additive polymorphisms detected in the ITS1 and ITS2 regions of*P. marginata*,*P. allionii*and*P. latifolia***

**Ploidy**	**Specie**	**Code**	**18**	**247**	**272**	**414**	**475**	**553**	**661**
6x	*P. latifolia*	**lat_1a**	C	A	-	T	C	C	A
6x		**lat_2a**	C	A	-	T	C	C	A
6x		**lat_3**	C	A	-	T	C	C	A
6x	*P. allionii*	**all_1**	C	A	A	T	T	C	S
6x		**all_2**	**M**	A	**T/-**	**Y**	C	C	C
6x	*P. marginata*	**mar_2**	A	S	**T/-**	**Y**	C	C	**M**
6x		**mar_3**	**M**	M	T	T	C	C	C
6x		**mar_6**	**M**	M	T	T	C	C	C
6x		**mar_7**	**M**	M	T	T	C	C	C
12x		**mar_10**	**M**	G	-	**Y**	**Y**	**Y**	A
12x		**mar_11**	**M**	S	**T/-**	**Y**	T	T	**M**
12x		**mar_13**	**M**	**V**	T	T	T	**Y**	C
12x		**mar_15**	**M**	M	T	C	C	C	C
12x		**mar_16**	**M**	M	**T/-**	**Y**	T	C	**M**
12x		**mar_17**	**M**	S	**T/-**	**Y**	**Y**	**Y**	**M**

Summary of additive polymorphisms (in bold) detected in the seven sites of the ITS1 and ITS2 regions of *P. marginata*, *P. allionii* and *P. latifolia:* ploidy levels, species names, population codes (as in Table 4), and positions of the seven sites in the aligned sequences are reported; ambiguity symbols according to IUPAC (Y = C or T, M = A or C, S = C or G, V = A or C or G). Dashes indicate a gap.

**Figure 4:**
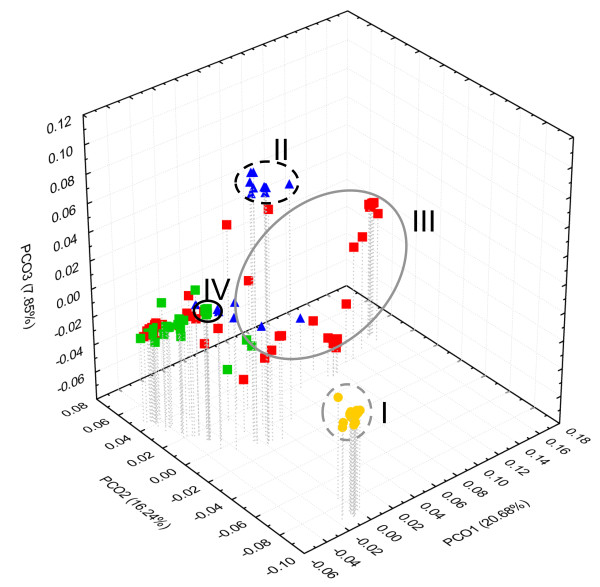
**PCoA scatterplot of ITS clones.** Scatterplot inferred from pairwise distances between cloned ITS sequences using the K2P substitution model: *P. marginata* hexaploids = green squares; *P. marginata* dodecaploids = red squares; *P. allionii* = blue triangles; *P. latifolia* = yellow circles. Ellipses indicate clades I-IV of Figure 3.

The three first principal coordinates of the PCoA explained 44.77% of the total molecular variation of the ITS clones. The results were consistent with the general structure of the ITS tree (Figure 4), for all sequences of *P. latifolia*, some sequences of *P. allionii*, and some sequences from *P. marginata* 12x samples formed three well to relatively well defined clusters, respectively; conversely, most other sequences from *P. allionii* and *P. marginata* hexaploids and dodecaploids clustered together.

The results of AMOVA (Table 3) indicated that most variation of the ITS clones is partitioned within individuals (values ranging between 69.91% and 62.30%), regardless of how the ITS sequences are grouped. Among groups, more variation is partitioned according to taxonomic classification, with (21.39% in H3) or without (24.01% in H2) cytological groupings in *P. marginata*, than according to the clades supported by the cpDNA phylogeny (4.33% in H1).

**Table 3:** Analysis of molecular variance (AMOVA) in cloned ITS sequences

**Hypotheses**	**Percentage of variation**		
	**among groups**	**among individuals within groups**	**within individuals**
H1	4.33^NS^	25.76*	69.91*
H2	24.01*	13.69*	62.30*
H3	21.39*	11.31*	67.30*

AMOVA was used to test whether variation was partitioned among groups that corresponded to: H1) the clades of the cpDNA phylogeny (i.e., *P. marginata/P. latifolia* clades and *P. allionii* clade); H2) current taxonomical classification (*P. marginata, P. latifolia* and *P. allionii*); H3) taxonomical classification with the subdivision of *P. marginata* in two cytotypes (*P. marginata* dodecaploids, *P. marginata* hexaploids, *P. latifolia* and *P. allionii*). * = P value < 0.01; ^NS^ = not significant.

The total nucleotide diversity of cloned ITS sequences was twice as high in *P. marginata* dodecaploids than in hexaploids (0.032 ± 0.016 vs. 0.015 ± 0.008, respectively). The Kendall Tau statistic did not show any significant correlation between the nucleotide diversity of all *P. marginata* individuals and the number of clones per individuals (r = −0.094; p > 0.05) nor number of individuals per population (r = −0.193; p > 0.05).

## Discussion

Pleistocene glacial cycles have been recognized as one of the major drivers of polyploid speciation in several alpine/arctic genera, including *CerastiumDrabaParnassiaSaxifraga* and *Vaccinium* [19; as reviewed in [37]. Polyploidization involving differentiated progenitors might have occurred in glacial refugia, where several species survived glacial maxima (e.g., *Androsace brigantiaca *[38]), or via secondary contact between populations that became isolated during glacial maxima and reconnected during interglacials [16,17], as proposed for *Primula* sect. *Aleuritia *[23].

The Pleistocene time frame for the evolution of *Primula* sect. *Auricula* inferred from both a section-wide ITS phylogeny [29] and a *Primula-*wide cpDNA phylogeny [De Vos & Conti, unpublished results] suggests that glacial cycles likely influenced the origin of *P. marginata*. In the present study, we use multiple lines of evidence to reconstruct the likely evolutionary history of *P. marginata* in relation to Pleistocene climate-dynamics. Our combined results allow us to propose possible superimposed processes that left distinctive signatures on the distribution of cytotypes and phylogenetic structure of the *P. marginata* chloroplast and nuclear genomes, suggesting events that might have occurred at different points in the evolution of the lineage. The complementarity of information between cytotype variation and nuclear and organellar genomes has proven indispensible to elucidate complex evolutionary histories in other boreal taxa, e.g. *Vaccinium *[39], *Artemisia *[40], and *Nymphaea *[41].

Flow cytometric analyses (Table 1) allowed us to expand the relatively limited, available information on cytotype variation (e.g., 41 individuals from 11 localities; [31,33,34]) to a survey of 100 individuals from 17 populations of *P. marginata*. Failure to detect any intermediate cytotypes nor any variation of ploidy levels within populations (Table 1) are congruent with the preliminary results of crossing experiments suggesting reproductive incompatibilities between the two cytotypes [Minuto et al., unpublished results]. Our investigations also enable us to confirm that the hexaploid and dodecaploid populations of *P. marginata* occur primarily in the western and eastern parts, respectively, of the species’ distributional range (Figure 2A).

Both adaptive and non-adaptive processes can explain the infra-population uniformity of cytotypes and their geographic separation. In the adaptive scenario, novel genetic combinations in the polyploids may allow them to adapt to different environmental conditions corresponding to specific geographic areas [7,13]. In the non-adaptive scenario, hybridization between cytotypes is thought to be either non-viable or produce plants with lower fitness, gradually leading to the elimination of the minority cytotype through frequency-dependent mechanisms (‘minority cytotype exclusion model’, [42]). These non-adaptive processes, while producing cytological uniformity, promote differentiation in a stochastic manner that does not usually produce distinct morphological and ecological characteristics [43,44]. In *P. marginata* the lack of morphological distinctiveness of the two cytotypes [25] and the absence of any obvious new ecological preferences in the dodecaploids [34] all fit the predictions of the non-adaptive scenario.

The two cytotypes of *P. marginata* do not form distinct clades either in the cpDNA or in the nrDNA phylogenies (Figures 2B, Figure 3), a pattern that seems more congruent with the stochastic nature of non-adaptive processes than with a scenario implying divergence between cytotypes adapted to distinct ecological conditions in different parts of the species range [13,42]. Despite the lack of reciprocal monophyly between the western, hexaploid and the eastern, dodecaploid populations, respectively, a signal of some geographic structure is detectable in the cpDNA topology. In this tree, the sequences of five hexaploid populations from the western part of the *P. marginata* range form a clade with a population of *P. latifolia* located further to the East (lat2; Figure 2B, clade 2), and a mixture of sequences from hexaploid and dodecaploid *P. marginata* populations eastward of the previously mentioned ones form a clade with two other samples of *P. latifolia* (Figure 2B, clade 1). The sharing of cpDNA haplotypes between *P. marginata* and *P. latifolia* might be explained by reticulation or incomplete lineage sorting from a polymorphic ancestor [45,46]. These processes are not necessarily mutually exclusive and are difficult to disentangle. However, assuming that the DNA sequences under exam are not under selection, a geographical pattern, as observed in our case, more likely results from introgression than lineage sorting [47].

In light of the non-adaptive scenario invoked above to explain the spatial separation between hexaploid and dodecaploid populations of *P. marginata* and the Pleistocene time frame for the evolution of *Primula* sect. *Auricula* [29; deVos, unpublished results], it is plausible to propose that abiotic processes, likely involving range fragmentation and/or shift during glacial maxima, contributed to the geographic signature in the cpDNA tree. More specifically, the clustering of all sampled dodecaploid populations of *P. marginata* with hexaploid populations from the southern portion of its distribution (Figure 2B clade 1), located in the glacial refugium of the Maritime Alps [48-50], might indicate that refugial populations from this area likely played a role in the origin of the dodecaploids. This explanation of geographic structure in the cpDNA phylogeny is consistent with the general interpretation of refugia on the southern side of the Alps as reservoirs of evolutionary potential during the climatic oscillations of the Pleistocene [29,38,48,50-54]. To summarize, the cpDNA tree indicates that, among the species of sect. *Auricula* that co-occur in the western Alps and are known to form hybrids with *P. marginata* either in nature or in cultivation [25,30], the latter shares a more recent common ancestor with *P. latifolia* than with *P. allionii* or *P. hirsuta,* while it excludes a close relationship with the allopatric *P. auricula *[33].

The nrDNA tree does not corroborate the common ancestry between *P. marginata* and *P. latifolia* supported by the cpDNA topology. The greater partitioning of ITS variation according to taxonomic groups (21.39% and 24.01%) than to the clades supported by the cpDNA phylogeny (4.33%; Table 3) provides a further indication of the topological conflict between the two phylogenies. In the nuclear phylogeny, all sequences from three populations of *P. latifolia* are included in a monophyletic group, while those of *P. marginata* and *P. allionii* are unresolved. Some sequences from a southern population of *P. allionii* (all2) share more recent common ancestors with sequences from hexaploid or dodecaploid populations of *P. marginata* (clades III, IV; Fig. 3) than with sequences from a northern population of *P. allionii* (all1), which form a well-supported clade (clade II; Figure 3). The non-monophyly of ribotypes from the same species may result from introgression between *P. marginata*-like and *P. allionii*-like ancestors, eventually leading to the homogenization of all2 sequences towards the *P. marginata* ribotypes. The interdigitation of nrDNA sequences in the phylogeny is reflected in the lack of distinct species clusters in the corresponding PCoA scatterplot (Figure 4). Congruently, most ITS variation is allocated within individuals (62.30-69.91%) rather than between groups in the AMOVA (Table 3). Our results also contradict the sister relationship between *P. marginata* and *P. latifolia* supported by one of 7770 equally parsimonious trees derived from direct sequencing of ITS amplicons in a previous study, where no cloning was performed [29]. In summary, nuclear ribotypes from different individuals of a *P. allionii* population located in the southern refugium of the Roya Valley in the Maritime Alps [49] and some hexaploid and dodecaploid populations of *P. marginata* coalesced more recently than haplotypes within the respective species, indicating a close and complex evolutionary history for their nuclear genomes.

Discrepancies between the phylogenetic signal of chloroplast and nuclear genomes have been found in several other polyploid complexes of the Northern hemisphere (e.g., *Vaccinium uliginosum *[39], *Primula* sect. *Aleuritia *[23], *Cerastium *[55], *Viola *[56]; see review in [19]). Explanations for cytonuclear conflicts range from differential substitution rates between the two genomes, hybridization/introgression, paralogy and incomplete homogenization or lineage sorting, especially of the nuclear sequences, or a combination of various processes [57-61]. Reciprocal illumination between different lines of evidence may help to favour some explanations over others. For instance, the neutral theory of molecular evolution [62] predicts that nuclear sequences should take longer to coalesce than organellar sequences, due to the larger, genetically effective population size of the former, especially in polyploid species [63,64]. Conversely, concerted evolution is supposed to speed the homogenization of multiple copies, as in the nrDNA region [59,65,66]. Another observation relevant to the interpretation of discrepancies between cpDNA and nrDNA phylogenies is that cpDNA sequences tend to evolve more slowly than ITS sequences, thus the former might be more suitable to track deeper evolutionary events, while the latter might better capture the phylogenetic signature of more recent events (e.g., [39,67]). Therefore, our phylogenetic results, taken together, seem compatible with the hypothesis of an initial homoploid introgression of the chloroplast genome between a *P. latifolia*-like ancestor and a *P. marginata* lineage, resulting in the persistence of cpDNA sequences that are more closely related to heterospecific than conspecific sequences (Figure 2B). The initial episode of chloroplast capture was probably followed by the separation between western and eastern populations of *P. marginata*, possibly driven by advancement of glaciers during glacial maxima, and the subsequent origin of the dodecaploids (Figure 2A). The occurrence of ITS clones from the all2 population of *P. allionii* in clades with dodecaploid and hexaploid individuals of *P. marginata* suggests that an ancestral southern population located in the Roya Valley, which was ice free in the Last Glacial Maximum [49], might have played a crucial role in the origin of *P. marginata* dodecaploids (Figure 2A, Figure 3). However, the lack of intermediate cytotypes in *P. marginata* (Table 1) and of any admixed individuals in an AFLP survey of *P. marginata* and *P. latifolia *[30] implies that the three species might have been evolutionarily isolated more recently, an interpretation also proposed by Kadereit et al. [30].

The higher proportion of ITS additive polymorphisms in dodecaploid than in hexaploid accessions of *P. marginata* (71% vs. 19.3%, respectively; Table 2) and higher total nucleotide diversity of ITS clones in the sequences of dodecaploid vs. hexaploid individuals (0.032 vs. 0.015) are congruent with an allopolyploid explanation for the origin of the dodecaploid populations of this primrose [68]. At the same time, the occurrence of shared additivity between *P. allionii* and *P. marginata* suggests a role of the former in the evolutionary history of the latter. The allopolyploid interpretation for the origin of *P. marginata* dodecaploids might appear to conflict with their lack of morphological differentiation [25,34; Casazza, unpublished observations]. Our case study might represent an example where the allopolyploids resemble one of the putative parental lineages, a situation that has been found also in *Mimulus *[69] and *Centaurea toletana *[70]; as reviewed in [10] and is compatible with the non-adaptive scenario [43,44] proposed above to explain the geographic separation between hexaploid and dodecaploid populations.

## Conclusions

Taken together, our results suggest that the dodecaploids of *P. marginata* are most likely of allopolyploid origin, as already proposed by Favarger [33], and that their origin is probably best interpreted within the context of glacial cycles during the Pleistocene, as proposed for the evolution of polyploids in *Primula* sect. *Aleuritia *[23]. While cpDNA introgression from a *P. latifolia-*like to a *P. marginata-*like lineage might have occurred earlier, subsequent hybridization events between southern refugial populations of hexaploid *P. allionii-*like and *P. marginata-*like ancestors were probably responsible for the origin of dodecaploid populations in the latter. Our study thus further underscores the important role of southern refugia, specifically in the Maritime Alps, as cradles of new biological diversity during the Pleistocene glaciations, as suggested also for other plant groups (e.g., *Carabus solieri *[52], *Saxifraga callosa *[71], *Androsace brigantiaca *[38]).

The adaptive vs. non-adaptive nature of the processes leading to the establishment of polyploids has different taxonomic consequences. In the adaptive scenario, the newly formed polyploids are adapted to specific ecological conditions and often display correspondingly distinct morphological characteristics, thus justifying the assignment of the different cytotypes to separate taxonomic units. Conversely, in the non-adaptive scenario, the frequent lack of any distinctive ecological or morphological features between cytotypes does not allow for any clear taxonomic differentiation. Therefore, the current evolutionary state of the hexaploid and dodecaploid cytotypes in *P. marginata* does not warrant taxonomic distinction, as already observed in other species (e.g., [43,44,68,72]). However, if the dodecaploids of *P. marginata* continue to remain reproductively isolated from the hexaploids of the same species and from *P. allionii*, as implied by the absence of intermediate cytotypes in their populations, over time they might differentiate from their relatives also morphologically, eventually leading to the origin of a taxonomically diagnosable species.

## Methods

### Sampling procedures

To investigate variation of ploidy levels in *P. marginata*, we collected three to eight individuals from each of 17 populations covering its entire distributional range (Table 4, Figure 2A). A total of 100 transplanted individuals were cultivated at the Botanical Garden of the University of Genova and later used for flow cytometry analyses at the Institute of Systematic Botany of the University of Zurich.

**Table 4:** **Sampling of*P. marginata*and co-occurring species of*Primula*sect.*Auricula*from the Western Alps**

** Species**	**locality**	**lat N**	**long E**	**code**	**Flowcytometry**	**cpDNA**	**nrDNA**
*P. allionii*	Vallone Scumbes, Val Gesso, Italy	44°17'22''	07°24'07''	**all1**		1	1
	Saint Dalmas de Tende, Vallée de la Roya, France	44°03'13''	07°35'26''	**all2**		1	1
	Chiarin, Vallée de la Roya, France	44°02'06''	07°33'34''	**all3**		1	
*P. hirsuta*	Rocca dell'Abisso, Val Vermenagna, Italy	44°08’24”	07°31’37"	**hir1**		1	
	Colle del Turlo, Val Sesia, Italy	45°54’09”	07°57’28”	**hir2**		1	
*P. latifolia*	Lago della Rovina, Val Gesso, Italy	44°10’25”	07°20’21”	**lat1a**		1	1
				**lat1b**		1	
	Riserva delle Navette, Val Tanarello, Italy	44°06’11"	07°43’04”	**lat2a**		1	1
				**lat2b**		1	
	Rocca dell'Abisso, Val Vermenagna, Italy	44°08’24”	07°31’37"	**lat3**		1	1
*P. marginata*	Chialvetta, Val Maira, Italy	44°26’56”	06°59'53"	**mar1**	5	1	
	Château Queyras, Vallée du Queyras, France	44°44’40”	06°46’11"	**mar2**	8	1	1
	Saint Auban, Vallée de l’Esteron, I’France	43°51’16”	06°43’29"	**mar3**	4	1	1
	Mont Bruna, Vallée de l’Esteron, I’France	43°54’41”	07°14’15"	**mar4**	6	1	
	Riofreddo, Valle Stura, Italy	44°17’39”	07°09’43”	**mar5**	5	1	
	L'Authion, Vallée de la Bevera, France	43°59’36”	07°25’36”	**mar6**	8	1	1
	Mont Baudion, Vallon de Paillon, France	43°48’02"	07°26’10"	**mar7**	6	1	1
	Cime du Grand Braus, Vallée de la Bevera, France	43°53’27”	07°29’20”	**mar8**	9	1	
	Beuil, Vallée du Var, France	44°06’00"	07°00’15"	**mar9**		1	
	Rocca Barbena, Valle del Neva, Italy	44°09’28”	08°07’39”	**mar10**	6	1	1
	Colle di Tenda, Val Vermenagna, Italy	44°09’15”	07°35’34”	**mar11**	5	1	1
	Monte Galero, Val Pennavaira, Italy	44°06'58”	07°55'30''	**mar12**	5	1	
	Monte Saccarello, Valle Tanaro, Italy	44°03’41”	07°42’52"	**mar13**	8	1	1
	Val Cravina, Val Pesio, Italy	44°13’48”	07°37'58”	**mar14**	5	1	
	Grotta del Bandito, Val Gesso, Italy	44°17’24”	07°25’44"	**mar15**	5	1	1
	Gorgie della Reina, Val Gesso, Italy	44°14'44''	07°25'41''	**mar16**	3	1	1
	Testa d'Alpe, Val Nervia, Italy	43°56’45"	07°33’28"	**mar17**	5	1	1
	Groppo Rosso, Val d'Aveto, Italy	44°33’43”	09°27’58"	**mar18**	7	1	
					100	28	15

Species, localities, coordinates (lat N, long E), population codes, and the number of individuals used for each kind of analysis (flow cytometry, cpDNA and nrDNA) are reported. All individuals collected in nature are cultivated at the Botanical Garden of the University of Genova. The 19 additional accessions for cpDNA phylogeny were reported in Additional file 1: Table S1.

To investigate the phylogenetic relationships of *P. marginata*, we collected and dried leaf tissue in silica gel from several populations of *P. marginataP. latifoliaP. allioniiP. hirsuta*, and related species in *Primula* sect. *Auricula*. To provide a broader phylogenetic context for the relationships of *P. marginata*, we also sampled 16 accessions from 15 species of sect. *Auricula* for the cpDNA analyses. Additionally, three taxa were selected for outgroup rooting based on published phylogenetic studies of *Primula *[29,73]: *P. cuneifolia* (sect. *Cuneifolia*) and *P. parryi* and *P. cusickiana* (sect. *Parryi*). Therefore, a total of 47 accessions were used for cpDNA analysis. A total of 15 accessions belonging to selected populations of *P. marginataP. allionii* and *P. latifolia* were used for nrDNA analysis (Table 4). Samples used in all analyses and GenBank/EBI accession numbers are listed in Table 4, Additional file 1: Tables S2, Additional file 1: Table S3,

### Flow cytometry

Flow-cytometric analyses were performed on fresh leaf tissue from a total of 100 individuals (Table 4). The haploid DNA content of *Pisum sativum* (1C = 4.88 pg, [74,75]) was used as internal standard. Approximately 1 cm^2^ of *P. marginata* leaf tissue was chopped together with the same quantity of *P. sativum* in 1ml of ice-cold Otto I buffer [76], modified as follows: 0.1 M citric acid, 0.5% Triton-X. The suspension was filtered through nylon mesh and centrifuged at 2000 RPM for 5 min. The pellet was resuspended in 40 μl of fresh Otto I buffer. The samples were stained with 160 μl of Otto II buffer (0.4 M Na_2_HPO_4_) supplemented with 1μg/μl DAPI (Sigma-Aldrich, Basel, Switzerland) for 20 min at room temperature and analyzed with the flow cytometer Cell Lab Quanta™ SC MPL (Beckman Coulter International S. A., Nyon, Switzerland). Isolated nuclei were excited by a Mercury arc UV lamp and the fluorescence intensity of 20000 nuclei was recorded. Ploidy levels were estimated according to the principle that peaks vary proportionally to the DNA content [77].

### DNA extraction, PCR amplification, cloning and sequencing

Total DNA was extracted from leaf tissue using the DNeasy Plant Mini Kit (Qiagen AG, Hombrechtikon, Switzerland), following the manufacturer’s instructions. The spacers *ndhF-rpl32psbD-trnTtrnD-trnT* of the cpDNA were amplified using primers (rpL32-R, ndhF; psbD, trnT^(GGU)^-R; trnT^(GGU)^, trnD^(GUC)^F) and protocols designed by Shaw et al. [78,79]. Amplification consisted of 5 min at 80°C followed by 30 cycles of: 1 min denaturation (95°C), 1 min annealing (50°C), followed by a ramp of 0.3°C/sec to 65°C, and 4 min extension (65°C). After the last cycle the temperature was kept at 65°C for the last 5 min of extension. A preliminary study that included two individuals per population of *P. marginata* detected no intra-population differences between the selected cpDNA target regions, therefore only one individual per population was included in subsequent cpDNA analyses.

The nuclear regions ITS1 and ITS2 were amplified using primers ITS-4 and ITS-Leu and protocols of Baum et al. [80]. Amplification reactions were performed in a total volume of 20 μl, adding 1 μl of DNA, 1x buffer (containing MgCl_2_), 2 mM MgCl_2,_ 0.2 mM dNTP’s, 0.2 μM of each primer, 1 unit of Taq Polymerase. The final concentration of MgCl_2_ was 3 mM. To decrease the formation of secondary structures and the amplification of pseudogenes, 5% of dimethyl sulfoxide (DMSO) was added to the amplification mixture, as recommended by Álvarez & Wendel [57]. Amplification consisted of 2 min at 94°C followed by 35 cycles of: 30 sec denaturation (94°C), 60 sec annealing (52°C), and 105 sec extension (72°C). After the last cycle the temperature was kept at 72°C for the last 10 min of extension and then lowered to 4°C.

To check for intra-individual variation of ITS repeats, we cloned ITS from one individual each of ten populations of *P. marginata*, three populations of *P. latifolia*, and two populations of *P. allionii* (Table 4). To reduce the risk of preferential amplification of ITS copies, a phenomenon known as PCR-drift or PCR-bias [81], the products of two PCR reactions per individual were combined and used for ligations. PCR products were column-cleaned with the NucleoSpin® Exctract II kit (Macherey-Nagel AG, Oensingen, Switzerland) and cloned using the CloneJET™ PCR Cloning Kit (Fermentas GmbH, Le Mont-sur-Lausanne, Switzerland), following the manufacturer’s instructions, but halving the recommended amounts of reagents. One Shot® MAX Efficiency® DH5α™-T1^R^ Competent Cells (Invitrogen AG, Basel, Switzerland) were used for transformation. The plasmid vector pJET was added to the cells, and these were shocked at 42°C for 30 seconds. After addition of 125 ml of LB medium, cells were incubated at 37°C for one hour and then spread on 50 μg/ml Ampicillin-agar plates. Twenty colonies per cloning reaction were selected to be PCR-amplified and then sequenced using the pJET1.2 primers.

All PCR products were cleaned by adding 1 μl of Calf Intestine Alcaline Phosphatase (CIAP) and 0.5 μl of Exonuclease I (Fermentas GmbH, Le Mont-sur-Lausanne, Switzerland) to each sample at 37°C for 15 min, followed by 15 min at 94°C to denature the enzymes. Cycle sequencing reactions were performed with the ABI Prism BigDye Terminator Cycle Sequencing Ready Reaction Kit (Applied Biosystems, Rotkreuz, Switzerland), using 1 μl of BigDye terminator per reaction. Cycle-sequencing products were purified on 96-well multiscreen filtration plates (Millipore, Zug, Switzerland) to remove unincorporated BigDye terminator and then analyzed on an ABI 3100 Genetic Analyzer (Applied Biosystems, Foster City, CA). The obtained sequences were edited and assembled using Sequencher 4.2 software (Gene Codes Corp., Ann Arbor, Michigan, USA).

### Alignment and phylogenetic analyses

All sequences of the three cpDNA regions were concatenated to form a single matrix. For the nrDNA matrix, the boundaries between the ITS1, 5.8S, and ITS2 sequences were determined by reference to *Rhododendron kanehirai* (GeneBank Z00044, [23]). Sequences in the cpDNA and nrDNA matrices were aligned with MUSCLE [82]; the resulting alignments were adjusted by eye in MacClade 4.06 [83]. Gaps were coded as binary characters with GapCoder [84] and added at the end of the cpDNA and nrDNA matrices. Ambiguous 1bp indels at the end of microsatellite regions were excluded from further analyses.

Phylogenies were inferred from the cpDNA and nrDNA matrices independently using both Maximum Parsimony (MP) and Bayesian Inference (BI). MP trees were inferred in PAUP 4.0b10 [85]. A heuristic search was performed with 100 random addition sequences, 10 trees held at each step, TBR branch swapping, MULTREES option off and “steepest descent” and “allswap” on. Maxtrees was set to 10000 with no autoincrease. Branch support was estimated with 1000 bootstrap replicates, using the TBR branch swapping algorithm and the same options as above.

For BI, the cpDNA matrix was partitioned into the three spacers while the nrDNA matrix was not partitioned. Models of nucleotide substitution were selected using an Akaike Information Criterion (AIC, [86]) in MrModeltest 2.3 [87]. The best model selected for the ITS matrix was the SYM + Γ model (Additional file 1: Table S4). For cpDNA spacers matrix, MrModeltest selected the GTR + Γ model for *psbD-trnT*, the GTR + I + Γ model for the *ndhF-rpl32*, and the HKY + I + Γ model for the *trnD-trnT*. Since the models selected for the two last regions could lead to parameter interaction, which might cause difficulty in reaching stationarity [88], we tested six different combinations of models (Additional file 1: Table S5) to choose the parameters that would best fit the data for these two genetic markers. Each analysis of the six different combinations was implemented in MrBayes v. 3.1.2 [89] on the Cipres Portal [90] and consisted of three to four runs of 10000000 generations from a random starting tree, with three heated and one cold chain, temperature set to 0.5, and trees sampled from the posterior probability distribution every 1000 generations. Convergence of the chains and stationarity were assessed in Tracer 1.5 [91], examining the plot of all parameter values and the log-likelihood against the number of generations. Stationarity was assumed when the standard deviation of the split frequencies was <0.01 and the Gelman & Rubin’s [92] convergence diagnostic (i.e., the potential scale reduction factor) was close to 1.0. The first 1000 trees of each Bayesian run were discarded as burnin, and the remaining trees in each analysis were used to calculate the posterior probabilities and 50% majority rule consensus tree. Bayes factors (BFs) were used to compare the models and choose the parameters that best fit the cpDNA data set [93]. BFs were calculated as the difference of the natural logarithm of the harmonic mean of the likelihood of the first model over the second model, multiplied by two [94]. BFs with positive values greater than ten were considered to provide very strong evidence against the alternative hypothesis. The best model combinations over all the six combinations tested was GTR + I for *ndhF-rpl32R* and HKY + Γ for *trnD-trnT* (Additional file 1: Table S4).

The topologies inferred from analyses of the cpDNA and nrDNA matrices were not identical. Several methods have been devised to estimate whether incongruence between trees is significant or not [95,96]. However, such tests could not be applied to our case, because they require the same number of terminals in both trees. Therefore, we performed a qualitative assessment of topological conflicts between the cpDNA and nrDNA trees by comparing posterior probabilities and bootstrap support values in both phylogenies (see also AMOVA test below).

### Additional analyses of cloned nrDNA sequences

A matrix including a total of 147 cloned nrDNA sequences was used to analyze the partitioning of infra-individual variation among nuclear ribotypes in *P. marginataP. latifolia*, and *P. allionii*. A polymorphic site in one accession was identified as additive when different nucleotides at the site were also found at the same site in at least one other accession in the data set [97]. If the polymorphism was found in one clone only, that site was not considered as additive, because it could be the product of Taq Polymerase misincorporation.

The structure of variation in the matrix of cloned nrDNA sequences was analyzed by Principal Coordinate Analysis (PCoA). We first calculated the pairwise distances in PAUP 4.0b10 [85] using the Kimura-2-Parameter (K2P) substitution model, then we calculated the axes values with the software SYN-TAX 2000 [98]. We drew the three-dimensional graphic using Statistica 8.0 [99].

The possible occurrence of recombinant sequences among the nrDNA clones was determined using the program RDP3 [100], which detects the most likely recombinants and their parental sequences, as well as the recombination breakpoints in the sequences. We compared the results of all seven methods implemented in the package (e.g., RDP [100], GENECONV [101], BOOTSCANNING [102], CHIMAERA [103], MAXCHI [104], SISCANN [105], 3SEQ [106]). Common settings for all methods were to consider sequences as linear, to require phylogenetic evidence, to disentangle overlapping signals, to polish breakpoints and to check alignment consistency. Statistical significance was set at the P = 0.05 level. Default options were used in each package, except same cases reported in Additional file 1: Table S6.

We performed an analysis of molecular variance (AMOVA) based on the K2P model of nucleotide substitution and 1000 iterations (Arlequin 3.11, [107]), to test whether ITS variation was partitioned among groups that corresponded to the following hypotheses: H1) the *P. marginata/P. latifolia* and *P. allionii* clades strongly supported by the cpDNA phylogeny; H2) current taxonomical classification (*P. marginata, P. latifolia* and *P. allionii*); H3) taxonomical classification with the subdivision of *P. marginata* in two cytotypes (*P. marginata* dodecaploids, *P. marginata* hexaploids, *P. latifolia* and *P. allionii*).

In order to discriminate between the potential autopolyploid vs. allopolyploid origin of dodecaplois in *P. marginata*, the nucleotide diversity [108] of cloned ITS sequences from 6x and 12x individuals were compared using Arlequin 3.11 [106]. According to Dobeš et al. [68], a nucleotide diversity approximately twice as high in polyploids indicates the involvement of distinct genetic lineages. To check for potential correlations between nucleotide diversity and number of clones per individual or the estimated number of individuals per population, we performed Kendal Tau correlation tests using Statistica 8.0 [99].

## Competing interests

The authors declare that they have no competing interests.

## Authors’ contributions

GC, EC and LM conceived the study. LG performed all lab work and analyses. GC, EC and LG provided crucial samples and contributed to data interpretation. GC, EC and LM wrote the paper. All authors read and approved the final manuscript.

## Supplementary Material

Additional file 1 **Table S1.** Sectional affiliations, species names, codes, and ploidy levels of species of sect. *Auricula* included in cpDNA phylogeny. **Table S2.** Species names, localities, codes and gene bank numbers of accessions of *Primula* sect. *Auricula* included in cpDNA phylogeny. **Table S3.** Species names, codes and GenBank/EBI accession numbers of *Primula allionii*, *P. latifolia* and *P. marginata* ITS clones included in nrDNA phylogeny. **Table S4.** Models of evolution, Base frequencies, and rates of substitutions estimated with MrModeltest for the different partitions used in this study. **Table S5.** Bayes factors across alternative models for two cpDNA data partitions. **Table S6.** Recombination analysis inferred among the nrDNA clones using the program RDP3. **Figure S1.** Maximum Parsimony 50% majority-rule consensus tree inferred from cpDNA sequences of *Primula* sect. *Auricula* accessions. **Figure S2.** Maximum Parsimony 50% majority-rule consensus tree of ITS clones. Click here for file
