# Supplementary material for: Polyploid evolution and Pleistocene glacial cycles: A case study from the alpine primrose Primula marginata (Primulaceae)
Source: BMC Evol Biol. 2012 Apr 24;12:56. doi: 10.1186/1471-2148-12-56 (PMC3444416; doi:10.1186/1471-2148-12-56)

Online Supplementary Material

Polyploid evolution and Pleistocene glacial cycles: a case study from the alpine primrose *Primula marginata* (Primulaceae)

Gabriele Casazza[1§], Laura Granato[1], Luigi Minuto[1] and Elena Conti[2]

[1]DIP.TE.RIS., University of Genoa, Corso Dogali 1M, I-16136, Genoa, Italy

[2]Institut für Systematische Botanik, Universität Zürich, Zollikerstrasse 107, CH-8008, Zürich, Switzerland

[§]Corresponding author

Email addresses:

GC: gabriele.casazza@unige.it

LG: laura.granato84@gmail.com

LM: minuto@dipteris.unige.it

EC: ContiElena@access.unizh.ch

6 Supplementary tables

2 Supplementary figures

TABLE S1. Sectional affiliations, species names, codes, and ploidy levels of species of sect. *Auricula* included in cpDNA phylogeny. Chromosome numbers are based on Kress [1,2] and Richards [3].

Section	Species	Code	Ploidy	References
Cuneifolia Balf.f.	*P. cuneifolia* Ledeb.	cuneif	2*x*	3
Parryi W.W.Sm. ex Wendelbo	*P. parryi* A.Gray	parryi	4*x*	3
	P. cusickiana (A.Gray) A.Gray	cusick	4*x?*	
Auricula Duby	*P. apennina* Widmer	apenn	6*x*	1,2
	P. auricula L.	auric	6*x*	1,2,3
	P. cottia Widmer	cottia	6*x*	1
	P. clusiana Tausch	clusia	18*x*	1,2,3
	P. daonensis (Leyb.) Leyb.	daone	6*x*	1,2,3
	P. deorum Velen.	deor1	6*x*	1,2,3
	P. deorum Velen.	deor2	6*x*	1,2,3
	P. glaucescens Moretti	glauc	6*x*	1,2,3
	P. glutinosa Wulfen in Jacq.	glutin	6*x*	1,2,3
	P. minima L.	minim	6*x*	1,2,3
	P. palinuri Petagna	palinu	6*x*	1,2,3
	P. pedemontana Thomas ex Gaudin	pedem	6*x*	1,2,3
	P. spectabilis Tratt.	spect	6*x*	1,2,3
	P. tyrolensis Schott	tyrol	6*x*	1,2,3
	P. villosa Wulfen in Jacq.	villosa	6*x*	1,2,3
	P. wulfeniana Schott	wulfen	6*x*	1,2,3

1. Kress A: **Zytotaxonomische untersucungen an den Primeln der section *Auricula* Pax.** *Österr Bot Z* 1963, 110:53-102.
2. Kress A: **Primulaceen-Studien, 10: Chromosomenzahlungen an verschiedenen Primulaceen**. Teil. C: *Primula*, Sectio *Auricula*. München: Gröbenzell; 1989.
3. Richards AJ: *Primula*. Portland, OR: Timber Press, 2003.

TABLE S2. Species names, localities, codes and gene bank numbers of accessions of *Primula* sect. *Auricula* included in cpDNA phylogeny.

**Species**	**Locality**	**Code**	**Voucher**	**ndhF-rpl32**	**psbD-trnT**	**trnD-trnT**
P. allionii Loisel.	Tetti La Crava, Vallon Saben, Val Gesso (CN), Italy	all1	HBGE	JQ755653	JQ755606	JQ755565
P. allionii Loisel.	San Dalmazzo di Tenda, Dogana, Val Roya, France	all2	HBGE	JQ755652	JQ755605	JQ755564
P. allionii Loisel.	Chiarin, Val Roya, Fance	all3	HBGE	JQ755651	JQ755604	JQ755563
P. hirsuta All.	Rocca dell'Abisso, Val Vermenagna, Italy	hir1	HBGE	JQ755627	JQ755583	JQ755542
P. hirsuta All.	Colle del Turlo, Val Sesia, Italy	hir2	HBGE	JQ755628	JQ755584	JQ755543
P. latifolia Lapeyr.	Lago della Rovina, Val Gesso, Italy	lat1a	HBGE	JQ755655	JQ755608	
P. latifolia Lapeyr.	Lago della Rovina, Val Gesso, Italy	lat1b	HBGE	JQ755650	JQ755603	JQ755562
P. latifolia Lapeyr.	Riserva delle Navette, Val Tanarello, Italy	lat2a	HBGE	JQ755654	JQ755607	
P. latifolia Lapeyr.	Riserva delle Navette, Val Tanarello, Italy	lat2b	HBGE	JQ755648	JQ755601	
P. latifolia Lapeyr.	Rocca dell'Abisso, Val Vermenagna, Italy	lat3	HBGE	JQ755649	JQ755602	JQ755561
P. marginata Curtis	Chialvetta, Val Maira, Italy	mar1	HBGE	JQ755639	JQ755592	JQ755552
P. marginata Curtis	Chateaux de Queiras, Vallée di Queiras, France	mar2	HBGE	JQ755643	JQ755596	JQ755556
P. marginata Curtis	Saint Auban, Vallée du , France	mar3	HBGE	JQ755632	JQ755586	
P. marginata Curtis	Mont Bruna, Vallée du , France	mar4	HBGE	JQ755647	JQ755600	JQ755560
P. marginata Curtis	Riofreddo, Valle Stura, Italy	mar5	HBGE	JQ755633	JQ755587	JQ755546
P. marginata Curtis	L'Authion, Vallée da Bevera, France	mar6	HBGE	JQ755631		JQ755545
P. marginata Curtis	Mont Baudion, Vallon de Paillon, France	mar7	HBGE	JQ755637	JQ755590	JQ755550
P. marginata Curtis	Cime de Grand Braus, Vallée da Bevera, France	mar8	HBGE	JQ755642	JQ755595	JQ755555
P. marginata Curtis	Beuil, M. Broussiere, Vallée du Var, France	mar9	HBGE	JQ755641	JQ755594	JQ755554
P. marginata Curtis	Rocca Barbena, Italy	mar10	HBGE	JQ755634		JQ755547

P. marginata Curtis	Cime du Bec Roux, Colle di Tenda, Val Vermenagna, Italy	mar11	HBGE	JQ755646	JQ755599	JQ755559
P. marginata Curtis	Monte Galero, M. della Guardia, Val Pennavaira, Italy	mar12	HBGE	JQ755635	JQ755588	JQ755548
P. marginata Curtis	Monte Saccarello, Valle Tanaro, Italy	mar13	HBGE	JQ755636	JQ755589	JQ755549
P. marginata Curtis	Val Cravina, Val Pesio, Italy	mar14	HBGE	JQ755640	JQ755593	JQ755553
P. marginata Curtis	Grotta del Bandito, Val Gesso, Italy	mar15	HBGE	JQ755645	JQ755598	JQ755558
P. marginata Curtis	Gorge de la Reina, Val Gesso, Italy	mar16	HBGE	JQ755644	JQ755597	JQ755557
P. marginata Curtis	Testa d'Alpe, Val Nervia, Italy	mar17	HBGE	JQ755630	JQ755585	
P. marginata Curtis	Groppo Rosso, Vla d'Aveto, Italy	mar18	HBGE	JQ755638	JQ755591	JQ755551
P. cuneifolia Ledeb.	Telaquana, Highlands (Turpnose Lake and Lake Clark	cuneif	Z	JQ755613	JQ755570	JQ755528
P. parryi A.Gray	A.R. Mast 720	parryi	Z	JQ755609	JQ755566	JQ755524
P. cusickiana (A.Gray) A.Gray	Boise, Idaho, USA, leg. S. Kelso, s.n.	cusick	COCO	JQ755612	JQ755569	JQ755527
P. apennina Widmer	Pendici N del M. Bozzo, Italy, leg. M. Ferrarini, 30 may 1993	apenn	FI	JQ755619	JQ755575	JQ755534
P. auricula L.	Arni di fronte alle Marmitte, Italy, leg. M. Ferrarini, 26 july 1991	auric	FI	JQ755618		JQ755533
P. cottia Widmer	Valle Faetto, Italy, leg. Pandolfo e Maglioni, 26/06/2008	cottia	TO	JQ755620	JQ755576	JQ755535
P. clusiana Tausch	Botanischen Garten-Graz, Austria; Steienmark, Priel Gruppe, Austria, ATOZ-20040936	clusia	HBZH	JQ755624	JQ755580	JQ755539
P. daonensis (Leyb.) Leyb.	Passo Gavia, Valtellina, Italy, leg. G. Pellegrino, 07 july 1988, s.n.	daone	Z	JQ755621	JQ755577	JQ755536
P. deorum Velen.	M. Rila, Maljovica, Bulgaria	deor1	B	JQ755610	JQ755567	JQ755525
P. deorum Velen.	M. Maljovica, Maljovica, Bulgaria, leg. J. Stepanek, 10 august 1997	deor2	B	JQ755611	JQ755568	JQ755526
P. glaucescens Moretti	Corno del Nibbio (LC), Italy, leg. G. Pellegrino, 06 may 1996, s.n.	glauc	Z	JQ755626	JQ755582	JQ755541
P. glutinosa Wulfen in Jacq.	A.R. Mast 708	glutin	Z	JQ755623	JQ755579	JQ755538
P. minima L.	Gruppo del Sella, Trentino, Italy, leg. G. Pellegrino, s.n.	minim	Z	JQ755625	JQ755581	JQ755540
P. palinuri Petagna	leg. Arne Anderberg SU-S-00.96.2	palinu	Z	JQ755629		JQ755544

P. pedemontana Thomas ex Gaudin	Colle del Nivoei, Val Soana, Italy, leg. G. Pellegrino, 10 july 1996, s.n.	pedem	Z	JQ755622	JQ755578	JQ755537
P. spectabilis Tratt.	Osterreichische Gartenbaugeseschaft-Graz, A; P.so Tremalzo, Alpi giudariche, Lombardia, Italy, ITOZ-20080304	spect	HBZH	JQ755617	JQ755574	JQ755532
P. tyrolensis Schott	Val Coalba verso Aia di Val Caldiera (TN), Italy, 1400 m, Roccette dolomitiche, Esp. N. leg. F. Prosser, 30 July 1998	tyrol	ROV	JQ755615	JQ755572	JQ755530
P. villosa Wulfen in Jacq.	Lago Mucrone (Oropa, BI), Italy, leg. G. Pellegrino, 11june 1989,s.n.	villosa	Z	JQ755616	JQ755573	JQ755531
P. wulfeniana Schott	Bundesgarten-Wien, Austria; Karntern, Barental, ATOZ-20050416	wulfen	HBZH	JQ755614	JQ755571	JQ755529

TABLE S3. Species names, codes and GenBank/EBI accession numbers of *Primula allionii*, *P. latifolia* and *P. marginata* ITS clones included in nrDNA phylogeny.

**Species**	**Code**	accessions numbers	**Species**	**Code**	accessions numbers
P. allionii Loisel.	all1_1	JQ755792	*P. marginata* Curtis	mar7_1	JQ755750
P. allionii Loisel.	all1_2	JQ755793	*P. marginata* Curtis	mar7_2	JQ755751
P. allionii Loisel.	all1_3	JQ755794	*P. marginata* Curtis	mar7_3	JQ755752
P. allionii Loisel.	all1_4	JQ755795	*P. marginata* Curtis	mar7_4	JQ755753
P. allionii Loisel.	all1_5	JQ755796	*P. marginata* Curtis	mar7_5	JQ755754
P. allionii Loisel.	all1_6	JQ755797	*P. marginata* Curtis	mar7_6	JQ755755
P. allionii Loisel.	all1_7	JQ755798	*P. marginata* Curtis	mar7_7	JQ755756
P. allionii Loisel.	all1_8	JQ755799	*P. marginata* Curtis	mar7_8	JQ755757
P. allionii Loisel.	all1_9	JQ755800	*P. marginata* Curtis	mar7_9	JQ755758
P. allionii Loisel.	all1_10	JQ755801	*P. marginata* Curtis	mar7_10	JQ755759
P. allionii Loisel.	all1_11	JQ755802	*P. marginata* Curtis	mar7_11	JQ755760
P. allionii Loisel.	all2_1	JQ755782	*P. marginata* Curtis	mar7_12	JQ755761
P. allionii Loisel.	all2_2	JQ755783	*P. marginata* Curtis	mar7_13	JQ755762
P. allionii Loisel.	all2_3	JQ755784	*P. marginata* Curtis	mar7_14	JQ755763
P. allionii Loisel.	all2_4	JQ755785	*P. marginata* Curtis	mar10_1	JQ755666
P. allionii Loisel.	all2_5	JQ755786	*P. marginata* Curtis	mar10_2	JQ755667
P. allionii Loisel.	all2_6	JQ755787	*P. marginata* Curtis	mar10_3	JQ755668
P. allionii Loisel.	all2_7	JQ755788	*P. marginata* Curtis	mar10_4	JQ755669
P. allionii Loisel.	all2_8	JQ755789	*P. marginata* Curtis	mar10_5	JQ755670
P. allionii Loisel.	all2_9	JQ755790	*P. marginata* Curtis	mar10_6	JQ755671
P. allionii Loisel.	all2_10	JQ755791	*P. marginata* Curtis	mar10_7	JQ755672
P. latifolia Lapeyr.	lat1a_1	JQ755717	*P. marginata* Curtis	mar10_8	JQ755673
P. latifolia Lapeyr.	lat1a_2	JQ755718	*P. marginata* Curtis	mar11_1	JQ755656
P. latifolia Lapeyr.	lat1a_3	JQ755719	*P. marginata* Curtis	mar11_2	JQ755657
P. latifolia Lapeyr.	lat1a_4	JQ755720	*P. marginata* Curtis	mar11_3	JQ755658
P. latifolia Lapeyr.	lat1a_5	JQ755721	*P. marginata* Curtis	mar11_4	JQ755659
P. latifolia Lapeyr.	lat1a_6	JQ755722	*P. marginata* Curtis	mar11_5	JQ755660
P. latifolia Lapeyr.	lat1a_7	JQ755723	*P. marginata* Curtis	mar11_6	JQ755661
P. latifolia Lapeyr.	lat1a_8	JQ755724	*P. marginata* Curtis	mar11_7	JQ755662
P. latifolia Lapeyr.	lat1a_9	JQ755725	*P. marginata* Curtis	mar11_8	JQ755663

P. latifolia Lapeyr.	lat2a_1	JQ755706	*P. marginata* Curtis	mar11_9	JQ755664
P. latifolia Lapeyr.	lat2a_2	JQ755707	*P. marginata* Curtis	mar11_10	JQ755665
P. latifolia Lapeyr.	lat2a_3	JQ755708	*P. marginata* Curtis	mar13_1	JQ755674
P. latifolia Lapeyr.	lat2a_4	JQ755709	*P. marginata* Curtis	mar13_2	JQ755675
P. latifolia Lapeyr.	lat2a_5	JQ755710	*P. marginata* Curtis	mar13_3	JQ755676
P. latifolia Lapeyr.	lat2a_6	JQ755711	*P. marginata* Curtis	mar13_4	JQ755677
P. latifolia Lapeyr.	lat2a_7	JQ755712	*P. marginata* Curtis	mar13_5	JQ755678
P. latifolia Lapeyr.	lat2a_8	JQ755713	*P. marginata* Curtis	mar13_6	JQ755679
P. latifolia Lapeyr.	lat2a_9	JQ755714	*P. marginata* Curtis	mar13_7	JQ755680
P. latifolia Lapeyr.	lat2a_10	JQ755715	*P. marginata* Curtis	mar13_8	JQ755681
P. latifolia Lapeyr.	lat2a_11	JQ755716	*P. marginata* Curtis	mar13_9	JQ755682
P. latifolia Lapeyr.	lat3_1	JQ755696	*P. marginata* Curtis	mar13_10	JQ755683
P. latifolia Lapeyr.	lat3_2	JQ755697	*P. marginata* Curtis	mar15_1	JQ755684
P. latifolia Lapeyr.	lat3_3	JQ755698	*P. marginata* Curtis	mar15_2	JQ755685
P. latifolia Lapeyr.	lat3_4	JQ755699	*P. marginata* Curtis	mar15_3	JQ755686
P. latifolia Lapeyr.	lat3_5	JQ755700	*P. marginata* Curtis	mar15_4	JQ755687
P. latifolia Lapeyr.	lat3_6	JQ755701	*P. marginata* Curtis	mar15_5	JQ755688
P. latifolia Lapeyr.	lat3_7	JQ755702	*P. marginata* Curtis	mar15_6	JQ755689
P. latifolia Lapeyr.	lat3_8	JQ755703	*P. marginata* Curtis	mar15_7	JQ755690
P. latifolia Lapeyr.	lat3_9	JQ755704	*P. marginata* Curtis	mar15_8	JQ755691
P. latifolia Lapeyr.	lat3_10	JQ755705	*P. marginata* Curtis	mar15_9	JQ755692
P. marginata Curtis	mar2_1	JQ755733	*P. marginata* Curtis	mar15_10	JQ755693
P. marginata Curtis	mar2_2	JQ755734	*P. marginata* Curtis	mar15_11	JQ755694
P. marginata Curtis	mar2_3	JQ755735	*P. marginata* Curtis	mar15_12	JQ755695
P. marginata Curtis	mar2_4	JQ755736	*P. marginata* Curtis	mar16_1	JQ755764
P. marginata Curtis	mar2_5	JQ755737	*P. marginata* Curtis	mar16_2	JQ755765
P. marginata Curtis	mar2_6	JQ755738	*P. marginata* Curtis	mar16_3	JQ755766
P. marginata Curtis	mar2_7	JQ755739	*P. marginata* Curtis	mar16_4	JQ755767
P. marginata Curtis	mar2_8	JQ755740	*P. marginata* Curtis	mar16_5	JQ755768
P. marginata Curtis	mar2_9	JQ755741	*P. marginata* Curtis	mar16_6	JQ755769
P. marginata Curtis	mar2_10	JQ755742	*P. marginata* Curtis	mar16_7	JQ755770
P. marginata Curtis	mar3_1	JQ755743	*P. marginata* Curtis	mar16_8	JQ755771
P. marginata Curtis	mar3_2	JQ755744	*P. marginata* Curtis	mar16_9	JQ755772
P. marginata Curtis	mar3_3	JQ755745	*P. marginata* Curtis	mar17_1	JQ755773
P. marginata Curtis	mar3_4	JQ755746	*P. marginata* Curtis	mar17_2	JQ755774

P. marginata Curtis	mar3_5	JQ755747	*P. marginata* Curtis	mar17_3	JQ755775
P. marginata Curtis	mar3_6	JQ755748	*P. marginata* Curtis	mar17_4	JQ755776
P. marginata Curtis	mar3_7	JQ755749	*P. marginata* Curtis	mar17_5	JQ755777
P. marginata Curtis	mar6_1	JQ755726	*P. marginata* Curtis	mar17_6	JQ755778
P. marginata Curtis	mar6_2	JQ755727	*P. marginata* Curtis	mar17_7	JQ755779
P. marginata Curtis	mar6_3	JQ755728	*P. marginata* Curtis	mar17_8	JQ755780
P. marginata Curtis	mar6_4	JQ755729	*P. marginata* Curtis	mar17_9	JQ755781
P. marginata Curtis	mar6_5	JQ755730			
P. marginata Curtis	mar6_6	JQ755731			
P. marginata Curtis	mar6_7	JQ755732			

TABLE S4. Models of evolution, Base frequencies, and rates of substitutions estimated with MrModeltest for the different partitions used in this study.

Data partition	Model	Base Frequency				Substitution rates (relative to G<->T rate)							
		A	C	G	T	A<->C	A<->G	A<->T	C<->G	C<->T	G<->T	I	г
ndhF-rpl32	GTR+I+г	0.4010	0.1103	0.0967	0.3921	0.8129	0.8680	0.2034	0.1561	0.6089	1	0.7107	0.8861
psbD-trnT	GTR+г	0.3479	0.1458	0.1327	0.3736	0.6090	0.3790	0.1769	0	0.6322	1		0.2775
trnD-trnT	HKY+I+г	0.3215	0.1736	0.1490	0.3559			Ti/Tv ratio = 0.6600				0.5280	0.8686
ITS	SYM+г		equal			2.1313	2.8728	1.0841	0.1745	3.3336	1		0.7594

TABLE S5. Bayes factors across alternative models for two cpDNA data partitions. Values above the diagonal show the Bayes factor support for model M1 over model M0. Values below the diagonal show Bayes factor support for M0 over M1.

M0 \ M1	ndhF-rpl32 GTR + I + Γ trnD-trnT HKY + Γ	ndhF-rpl32 GTR + I trnD-trnT HKY + Γ	ndhF-rpl32 GTR + Γ trnD-trnT HKY + Γ	ndhF-rpl32 GTR + I + Γ trnD-trnT HKY + Γ	ndhF-rpl32 GTR + I trnD-trnT HKY + Γ	ndhF-rpl32 GTR + Γ trnD-trnT HKY + Γ
ndhF-rpl32 GTR + I + Γ trnD-trnT HKY + Γ	/	-2.158	-0.34	155.662	160.574	148.500
ndhF-rpl32 GTR + I trnD-trnT HKY + Γ	2.158	/	1.818	157.820	162.732	150.658
ndhF-rpl32 GTR + Γ trnD-trnT GTR + Γ	0.340	-1.818	/	156.002	160.914	148.84
ndhF-rpl32 GTR + I + Γ trnD-trnT HKY + I + Γ	-155.662	-157.82	-156.002	/	4.912	-7.162
ndhF-rpl32 GTR + I trnD-trnT HKY + I + Γ	-160.574	-162.732	-160.914	-4.912	/	-12.074
ndhF-rpl32 GTR + Γ trnD-trnT HKY + I + Γ	-148.500	-150.658	-148.84	7.162	12.074	/

TABLE S6. Recombination analysis inferred among the nrDNA clones using the program RDP3. Default options were used, except in the following cases. In the RDP method, the “no reference” option was used, because it is recommended in matrices with more than 50 sequences that are not too divergent from each other (more than 60% identity). In the GENECONV method, indels were interpreted as single polymorphisms. For BOOTSCAN, a window-size of 80 and a step size of 20 were chosen, in order to meet the expectation of having at least 10 polymorphic sites in a single window; the option “neighbour-joining trees” was used to determine the relationships between sequences and 500 bootstrap replicates with a 95% cutoff value were performed, calculating binomial P-values. For SISCAN the same step and window-size as in the BOOTSCAN method was used, and 100 permutations were performed.

	Breakpoint Positions									Detection Methods and P-value						
	In Alignment		In Recombinant Sequence		Relative to mar11_1											
Recombination Event Number	Begin	End	Begin	End	Begin	End	Recombinant Sequence(s)	Minor Parental Sequence(s)	Major Parental Sequence(s)	RDP	GENECONV	Bootscan	Maxchi	Chimaera	SiSscan	3Seq
1	334*	632	319*	613	319*	612	mar2_6	Unknown (mar2_9)	mar2_7	NS	NS	NS	NS	NS	NS	2.89E+12
1							mar2_1[T]	mar2_2								

* = The actual breakpoint position is undetermined (it was most likely overprinted by a subsequent recombination event).

Minor Parent = Parent contributing the smaller fraction of sequence.

Major Parent = Parent contributing the larger fraction of sequence.

Unknown = Only one parent and a recombinant need be in the alignment for a recombination event to be detectable.

The sequence listed as unknown was used to infer the existence of a missing parental sequence.

NS = No significant P-value was recorded for this recombination event using this method.

SUPPLEMENTARY FIGURES

FIGURE S1. Maximum Parsimony 50% majority-rule consensus tree inferred from cpDNA sequences of *Primula* sect. *Auricula* accessions. Bootstrap support (BS) values are indicated above the branches. *P. marginata* hexaploids = green squares; *P. marginata* dodecaploids = red squares; *P. allionii* = blue triangles; *P. latifolia* = yellow circles *P. hirsuta* = pink diamond. Population codes as in Table 1 and Supplementary Table S1.

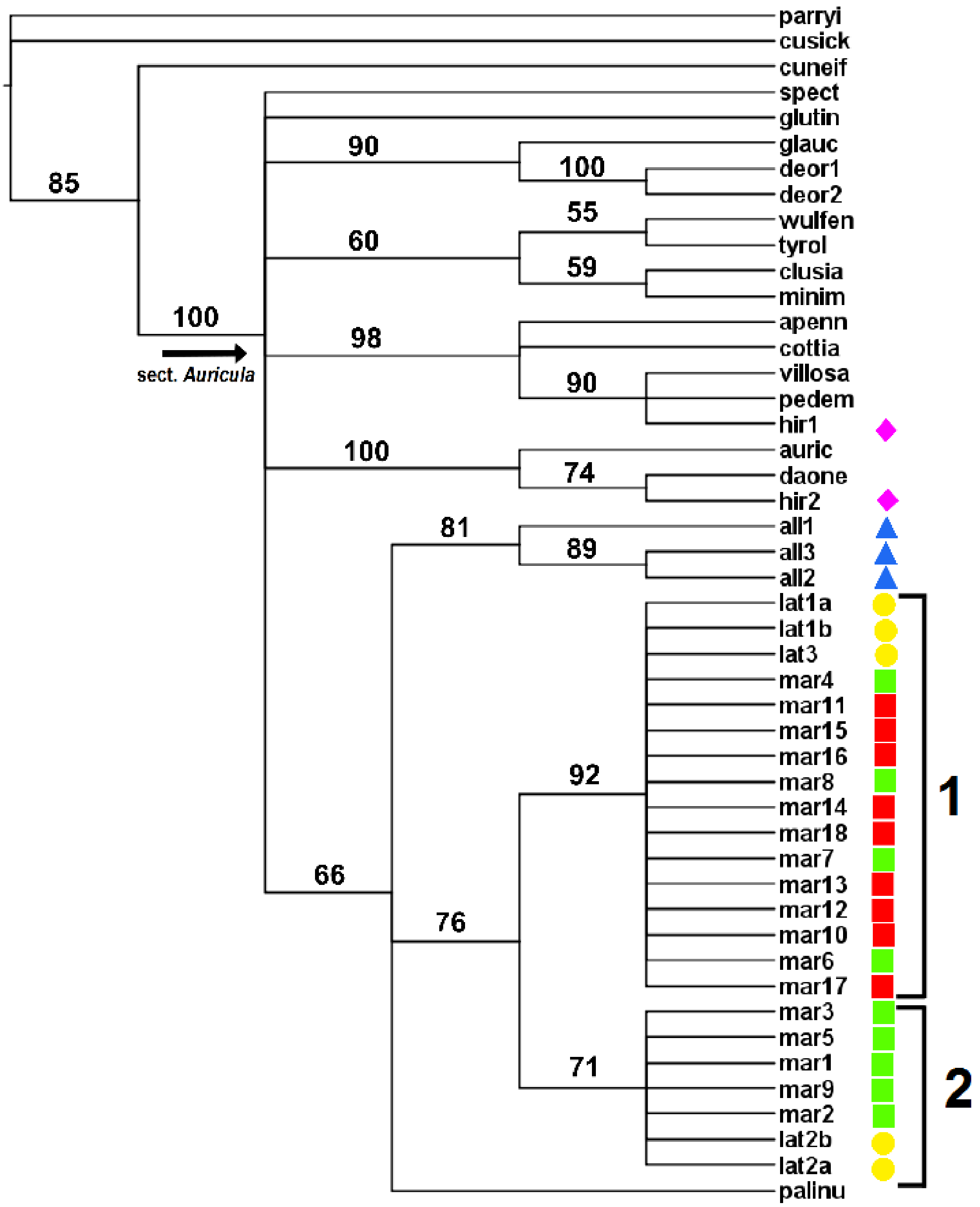

FIGURE S2. Maximum Parsimony 50% majority-rule consensus tree of ITS clones. Bootstrap support (BS) values are indicated above the branches. The colors distinguish species and ploidy levels: *P. marginata* hexaploids = green. *P. marginata* dodecaploids = red. *P. allionii* = blu. *P. latifolia* = yellow. Accessions codes as in Table 1; clones are numbered progressively for each accession.

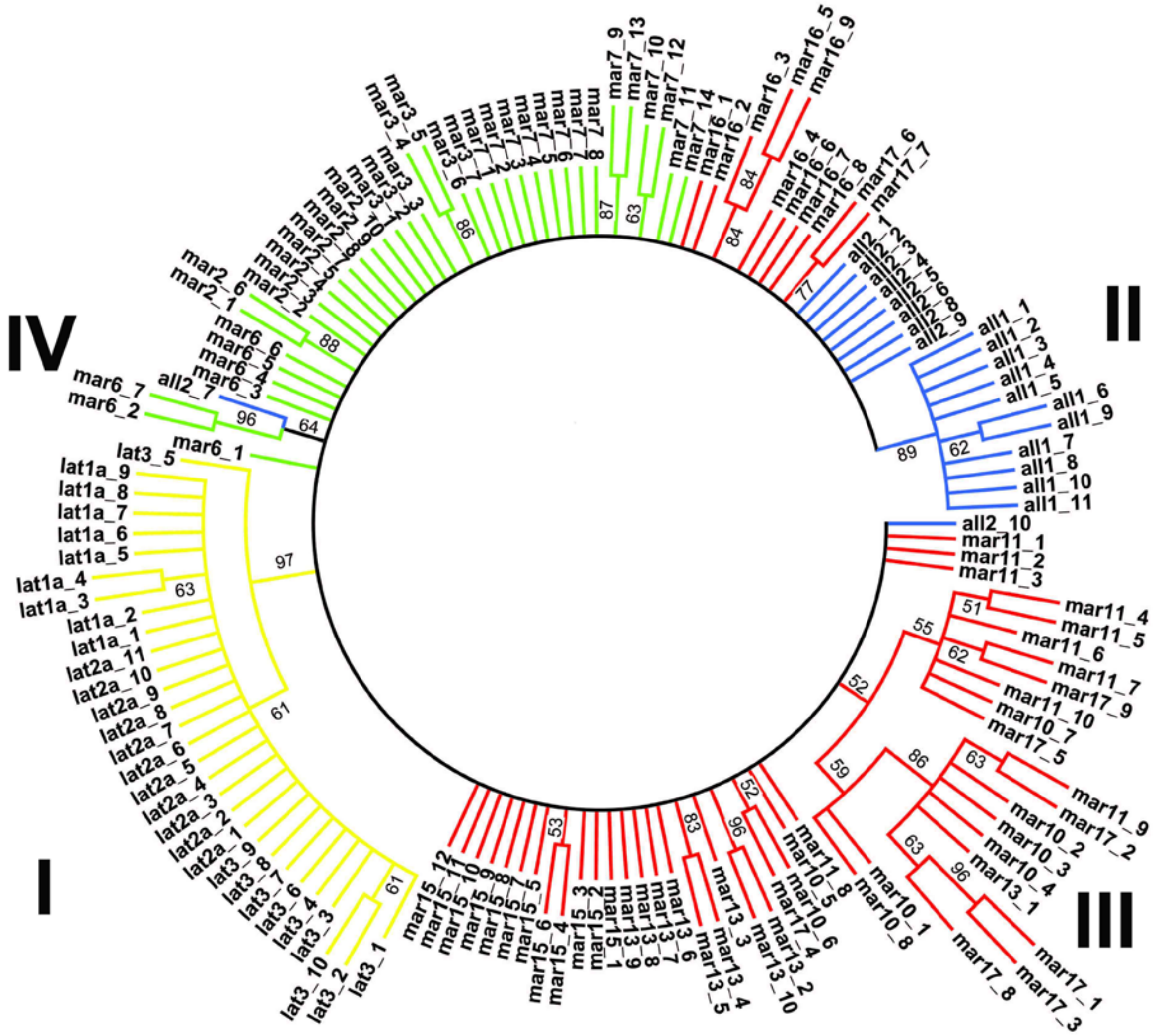

Supplement: Additional file 1 — Table S1. Sectional affiliations, species names, codes, and ploidy levels of species of sect. Auricula included in cpDNA phylogeny. Table S2. Species names, localities, codes and gene bank numbers of accessions of Primula sect. Auricula included in cpDNA phylogeny. Table S3. Species names, codes and GenBank/EBI accession numbers of Primula allionii, P. latifolia and P. marginata ITS clones included in nrDNA phylogeny. Table S4. Models of evolution, Base frequencies, and rates of substitutions estimated with MrModeltest for the different partitions used in this study. Table S5. Bayes factors across alternative models for two cpDNA data partitions. Table S6. Recombination analysis inferred among the nrDNA clones using the program RDP3. Figure S1. Maximum Parsimony 50% majority-rule consensus tree inferred from cpDNA sequences of Primula sect. Auricula accessions. Figure S2. Maximum Parsimony 50% majority-rule consensus tree of ITS clones. [file 1471-2148-12-56-S1.pdf]
